# Developmental Programming and Reprogramming of Hypertension and Kidney Disease: Impact of Tryptophan Metabolism

**DOI:** 10.3390/ijms21228705

**Published:** 2020-11-18

**Authors:** Chien-Ning Hsu, You-Lin Tain

**Affiliations:** 1 Department of Pharmacy, Kaohsiung Chang Gung Memorial Hospital, Kaohsiung 833, Taiwan; chien_ning_hsu@hotmail.com; 2 School of Pharmacy, Kaohsiung Medical University, Kaohsiung 807, Taiwan; 3 Department of Pediatrics, Kaohsiung Chang Gung Memorial Hospital and Chang Gung University College of Medicine, Kaohsiung 833, Taiwan; 4 Institute for Translational Research in Biomedicine, Kaohsiung Chang Gung Memorial Hospital and Chang Gung University College of Medicine, Kaohsiung 833, Taiwan

**Keywords:** aryl hydrocarbon receptor, chronic kidney disease, developmental origins of health and disease (DOHaD), hypertension, indole, kynurenine, melatonin, serotonin, tryptophan, uremic toxin

## Abstract

The concept that hypertension and chronic kidney disease (CKD) originate in early life has emerged recently. During pregnancy, tryptophan is crucial for maternal protein synthesis and fetal development. On one hand, impaired tryptophan metabolic pathway in pregnancy impacts fetal programming, resulting in the developmental programming of hypertension and kidney disease in adult offspring. On the other hand, tryptophan-related interventions might serve as reprogramming strategies to prevent a disease from occurring. In the present review, we aim to summarize (1) the three major tryptophan metabolic pathways, (2) the impact of tryptophan metabolism in pregnancy, (3) the interplay occurring between tryptophan metabolites and gut microbiota on the production of uremic toxins, (4) the role of tryptophan-derived metabolites-induced hypertension and CKD of developmental origin, (5) the therapeutic options in pregnancy that could aid in reprogramming adverse effects to protect offspring against hypertension and CKD, and (6) possible mechanisms linking tryptophan metabolism to developmental programming of hypertension and kidney disease.

## 1. Introduction

Hypertension affects more than one fourth of the global population [1]. Despite pharmacotherapy advances over the past decades, the prevalence of hypertension is still rising globally [2]. Like hypertension, chronic kidney disease (CKD) is another major public health concern around the world [3]. Approximately 10% of the population worldwide is affected by CKD. Hypertension and CKD are bidirectionally interlinked, because aspects of the pathophysiology are shared by both diseases in the kidneys. Hypertension is a risk factor for CKD and most patients with CKD have hypertension. Although hypertension and CKD are more common in adults, both of which can be driven by environmental insults in early life [4,5]. This has given rise to the concept of “developmental origins of health and disease” (DOHaD) [6]. Maternal nutrition is an important factor which determines fetal development. Imbalanced maternal nutrition during pregnancy and lactation produces fetal programming that permanently alter the body’s morphology and function and leads to many adult diseases, including hypertension and CKD [7]. Adverse renal programming alters structure and function of the kidneys permanently and increases the risk for developing hypertension and kidney disease later in life [8,9]. Conversely, early-life nutritional interventions have recently started to gain importance to reverse the programming processes to prevent hypertension and CKD by so-called reprogramming [10].

Tryptophan is a nutritionally essential amino acid that must be provided through dietary sources. Given the complexity of tryptophan metabolic pathways, the diverse properties of tryptophan-derived metabolites have been linked to various pathophysiological states [11,12,13]. Additionally, tryptophan metabolism has emerged as a central hub for host–microbe interactions [14]. During pregnancy, tryptophan is mandatory because of increased demand for maternal protein synthesis and fetal growth and development [15]. Although the estimated average requirement (EAR) in pregnancy for total protein is recommended [16], there remains a lack of recommendation of a specific amino acid like tryptophan for pregnant women. Endogenous tryptophan metabolites (e.g., serotonin and melatonin) and microbial tryptophan catabolites (e.g., indole and indole-3-aldehyde) have been linked to hypertension and kidney disease [17,18]; however, the impact of maternal tryptophan metabolism on the development of hypertension and kidney disease in adult offspring is still largely unknown.

According to the two aspects of the DOHaD concept, tryptophan metabolic pathways may act as a double-edged sword in developmental programming of hypertension and CKD. This review, therefore, highlights evidence on the impact of tryptophan metabolism during pregnancy on offspring hypertension and kidney disease, as well as the role of tryptophan-related interventions as a reprogramming strategy in the prevention of hypertension of CKD in adult offspring.

We searched the PubMed/MEDLINE databases for studies published in English between January 1990 and July 2020, using the following search terms: “blood pressure,” ”chronic kidney disease” “developmental programming,” “DOHaD,” “gestation” “hypertension,” “indole,” “indoxyl sulfate,” “kynurenine,” “melatonin,” “mother,” “maternal,” “offspring,” “progeny,” “pregnancy,” “perinatal,” “serotonin,” and “tryptophan.” Relevant studies were assessed for inclusion by combining the title and abstract screening, followed by a review of full-text studies.

## 2. Tryptophan Metabolism

### 2.1. Tryptophan Metabolic Pathways

**Figure 1:**
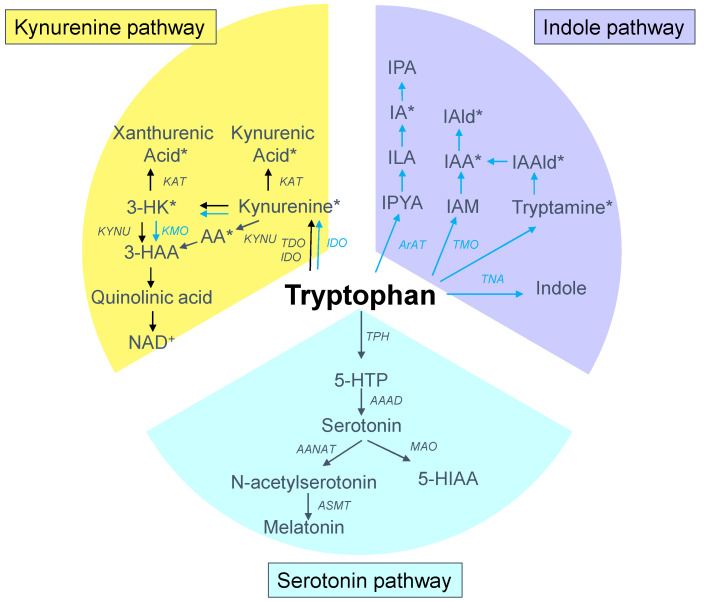
Overview of tryptophan metabolism through the kynurenine (yellow), serotonin (blue), and indole (purple) metabolic pathways. The black arrow lines indicate the host pathway, while the blue arrow lines indicate the microbial pathway. The asterisk indicates the aryl hydrocarbon receptor (AhR) ligand. IDO = indoleamine 2-3-dioxygenase; TDO = tryptophan 2,3-dioxygenase; AA = anthranilic acid; KMO = kynurenine-3-monooxygenase; KYNU = kynureninase; 3-HK = 3-hydroxykynurenine; KAT = kynurenine aminotransferase (KAT); 3-HAA = 3-hydroxyanthranilic acid; NAD^+^ = nicotinamide adenine dinucleotide; TPH = tryptophan hydroxylase; 5-HTP = 5-hydroxytryptophan; AAAD = aromatic amino acid decarboxylase; AANAT = arylalkylamine N-acetyltransferase; ASMT = N-acetylserotonin O-methyltransferase; MAO = monoamine oxidase; 5-HIAA = 5-hydroxyindoleacetic acid; TNA = tryptophanase; IAA = indoleacetic acid; IAld = indole-3-aldehyde; IAAld = indole-3-acetaldehyde (IAAld); IA = indoleacrylic acid; IPA = indole-3-propionic acid; ILA = indolelactic acid; IPYA = Indole-3-pyruvate; ArAT = acromatic amino acid aminotransferase; TMO = tryptophan 2-monooxygenase; IAM = indole-3-acetamide.

Tryptophan is present in protein-based foods, particularly meat, milk, peanuts and fish [11]. Approximately one-third of the whole-body flux of tryptophan comes from a dietary source, and the rest is from protein degradation. Although tryptophan is found in the smallest concentrations of the 20 amino acids in the human body [11], it has complex and multifaceted biological effects due to its wide range of biologically active metabolites along various metabolic pathways. Tryptophan metabolism follows three major pathways in the gut: (1) the kynurenine pathway in both epithelial and immune cells; (2) the serotonin pathway in enterochromaffin cells; and (3) the indole pathway by the gut microbiota [14]. A schematic summarizing the major tryptophan metabolic pathways is presented in Figure 1. Tryptophan absorption is primarily mediated by the solute carrier (SLC) 6A19, encoding system B (0) neutral amino acid transporter 1 (B^0^AT1). Over 95% of the absorbed tryptophan is catabolized via the kynurenine pathway, while only 1–2% and 2–3% of dietary tryptophan are converted into serotonin and indole pathways, respectively [19,20].

First, the kynurenine pathway plays a critical role in generating cellular energy in the form of nicotinamide adenine dinucleotide (NAD^+^) [20]. The initial and rate-limiting step in the kynurenine pathway involves one of three enzymes, namely, the indoleamine 2-3-dioxygenase 1 and 2 (IDO1 and IDO2) and tryptophan 2,3-dioxygenase (TDO). The product of the IDO/TDO-catalyzed reaction, N-formylkynurenine, is then hydrolyzed to kynurenine. Following its synthesis, kynurenine can be further metabolized by various enzymes. Kynureninase (KYNU) produces anthranilic acid (AA) from kynurenine. Kynurenine-3-monooxygenase (KMO) converts KYN into 3-hydroxykynurenine (3-HK), which can be taken by kynurenine aminotransferase (KAT) to produce xanthurenic acid or by the KYNU to form 3-hydroxyanthranilic acid (3-HAA). Catabolism of 3-HAA can lead to the generation of picolinic acid, quinolinic acid, and NAD^+^. In addition, KAT can metabolize kynurenine into kynurenic acid.

Second, the serotonin pathway is initiated by tryptophan being hydroxylated by tryptophan hydroxylase (TPH) to the intermediate 5-hydroxytryptophan (5-HTP), which is subsequently decarboxylated by aromatic amino acid decarboxylase (AAAD) to become serotonin (5-hydroxytryptpamine, 5-HT). The enterochromaffin cells in the gut account for almost 90% of the human body’s serotonin synthesis. Serotonin can be acetylated to form N-acetylserotonin by arylalkylamine N-acetyltransferase (AANAT), followed by N-acetylserotonin O-methyltransferase (ASMT) to generate melatonin. One the other hand, serotonin can be metabolized by monoamine oxidase (MAO) to 5-hydroxyindoleacetic acid (5-HIAA).

**Figure 2:**
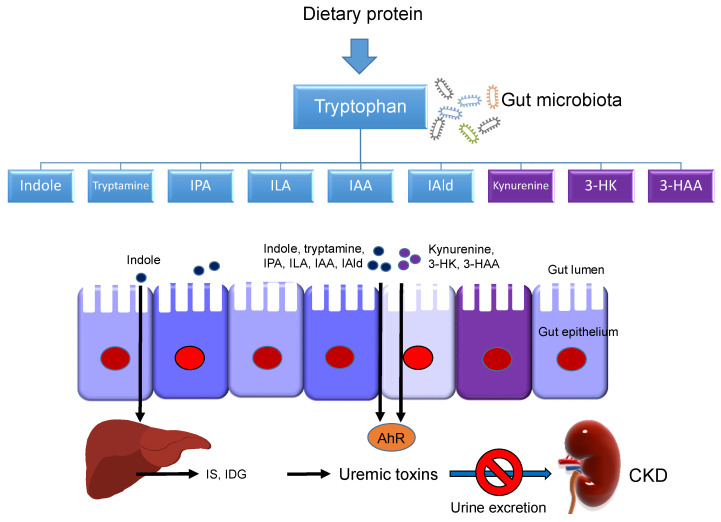
Schematic representation of the interplay occurring between tryptophan metabolites and gut microbiota on the production of uremic toxins in patients with chronic kidney disease (CKD). Microbial tryptophan catabolites are from the kynurenine (purple box) and indole (blue box) metabolic pathways. The black arrow lines indicate tryptophan metabolites are absorbed through the gut epithelium and enter the bloodstream. The indole can be further metabolized to indoxyl sulfate (IS) and indoxyl-β-D glucuronide (IDG) in the liver. Under chronic kidney disease (CKD), the kidneys are unable to excrete these tryptophan metabolites and cause the accumulation of uremic toxins. AhR = aryl hydrocarbon receptor; 3-HK = 3-hydroxykynurenine; 3-HAA = 3-hydroxyanthranilic acid; IPA = indole-3-propionic acid; ILA = indolelactic acid; IAA = indoleacetic acid; IAld = indole-3-aldehyde.

Last, a small amount of tryptophan is converted by gut microbiota into tryptamine, and through the action of the enzyme tryptophanase (TNA) into indole and its derivatives [13]. Tryptamine induces the release of serotonin by enterochromaffin cells. Indoles can be further metabolized to indoxyl sulfate (IS) and indoxyl-β-D glucuronide (IDG) in the liver [21]. Many indole derivatives, such as indoleacetic acid (IAA), indole-3-aldehyde (IAld), indole-3-acetaldehyde (IAAld), indoleacrylic acid (IA), and indolelactic acid (ILA), are ligands for aryl hydrocarbon receptor (AhR) [22]. As shown in Figure 2, several microbial tryptophan catabolites like IS, IDG, and IAA are unable to be excreted in urine in patients with CKD. These tryptophan metabolites are accumulated as uremic toxins [23]. In addition to the indole pathway, tryptophan-derived uremic toxins can also come from the kynurenine pathway, such as kynurenine, kynurenic acid, 3-HK, 3-HAA, and quinolinic acid.

### 2.2. Tryptophan Metabolism in Pregnancy

The quantitative dietary tryptophan requirement varies broadly across species [24]. The recommended daily intake of tryptophan was 4 mg/kg body weight for adult humans by the WHO [25]. Tryptophan requirements in pregnancy are increased, because of the increased demand for maternal protein synthesis, fetal requirements for growth and development, serotonin release for signaling pathways, production of KA for neuronal protection, and NAD^+^ synthesis [15]. Previous studies illustrated the increased requirement and transport of tryptophan to the fetus because tryptophan level in umbilical cord was higher than in maternal plasma [15]. Pregnancy-associated plasma hypoaminoacidemia develops early in gestation and persists throughout pregnancy [26]. Whole-body protein turnover studies demonstrated increased protein synthesis by 15% in the second trimester and 25% during the third trimester [25]. The current recommended pregnancy EAR for total protein is 0.88 g/kg/day, which is 1.33 times the EAR for non-pregnant adults; however, no recommendation has been developed for tryptophan requirements in human pregnancy [27]. In swine, the requirements for tryptophan have been revealed to increase by 35% during the late stages of pregnancy when compared to the early stages [28]. However, gestation-stage-specific dietary tryptophan recommendation awaits further evidence.

Excessive or insufficient consumption of a specific amino acid has been linked to adverse fetal outcomes [29,30]. Plasma tryptophan level is high at birth, but quickly declines within 24 h and reaches normal values by day 7 of life [31]. In terms of placentas at delivery, the KYN-to-tryptophan ratio measured in the chorionic plate is higher than that in the peripheral blood, suggesting placental IDO activity is highly active at delivery [32]. Additionally, the NAD pathway is enhanced in pregnant women and rats [33]. In the serotonin pathway, an adequate supply of serotonin and melatonin is crucial for fetal development [34,35,36]. Clinical and experimental evidence support the notion that perinatal selective serotonin reuptake inhibitors (SSRI) exposure can reduce body weight, impair brain development, and cause life-long adverse emotional health [37]. In a maternal melatonin deficient rat model, offspring had disrupted circadian rhythms and intrauterine growth retardation (IUGR), which were prevented by maternal melatonin treatment [38]. These findings indicate that tryptophan-derived metabolites in the serotonin pathway acts in different ways in the maternal–fetal system to bring on a successful pregnancy and fetal development.

## 3. Tryptophan Metabolism in Hypertension and Kidney Disease

**Figure 3:**
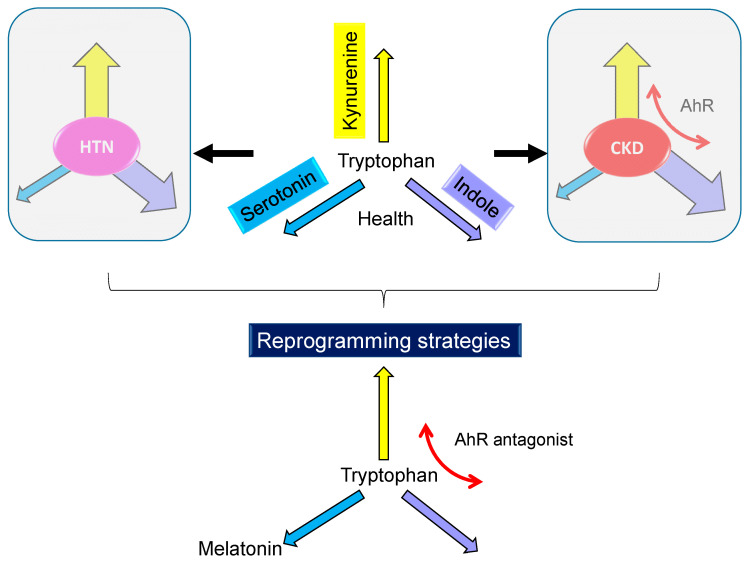
Schematic diagrams indicate repartitioning of tryptophan metabolic pathways in hypertension (HTN) and chronic kidney disease (CKD) based on the available literature data. The three major tryptophan metabolic pathways are kynurenine (yellow), serotonin (blue), and indole (purple) pathways. They are tightly interconnected to maintain good health and differentially affected in diseases. Weights of arrow lines indicate strength of pathway activation. The restoration of impaired tryptophan metabolic pathways using tryptophan supplementation, melatonin, or aryl hydrocarbon receptor (AhR) antagonist represents a promising reprogramming strategy.

Dietary tryptophan was shown to have a negative correlation with systolic BP in the TwinsUK study [39], while this finding was not supported by other evidence [40]. In hypertensive animal models, dietary tryptophan has been reported to attenuate the development of hypertension in the spontaneously hypertensive rats [41], Dahl salt-sensitive rats [42], and renovascular hypertensive rats [43]. Of note is that not only tryptophan but also its metabolites have vasodilatory property [44]. First, KYN was known to dilate coronary arteries or aorta in different animal species in a dose-dependent manner [44]. In a renovascular hypertensive rat model, higher plasma levels of several kynurenine metabolites such as KYN and AA were found in hypertensive rats than in sham rats [45]. In contrast, overexpression of IDO, a rate-limiting enzyme in the kynurenine pathway, in endothelium could protect against hypoxia-induced pulmonary hypertension in rodents [46]. These observations suggest hypertension is associated with activation of the kynurenine pathway (Figure 3). Second, the role played by serotonin in BP regulation is complex and still unclear [47]. Variations of serotonin levels in the plasma and platelet in different types of hypertensive patients can be increased [48], unaltered [49], or even decreased [50]. Although human and animal studies showed serotonin mainly results in acute and direct effect of arterial constriction [47], chronic administration of serotonin causes a long-term decrease in BP [51,52]. Melatonin is another important metabolite in the serotonin metabolic pathway. As we reviewed elsewhere, melatonin can prevent the development of hypertension via receptor-dependent and receptor-independent actions [53].

Uremic toxins derived from tryptophan fermentation by gut microbiota are associated with cardiovascular disease (CVD) in patients with CKD [17,23]. Tryptophan-derived uremic toxins, mainly coming from the indole and kynurenine pathways, have prooxidant, proinflammatory, procoagulant, and pro-apoptotic effects [23]; moreover, most of them are potent AhR ligands [23]. In patients with CKD, serum tryptophan level is decreased whereas metabolites of the kynurenine pathway are increased [54,55]. Despite the fact that hemodialysis can reduce kynurenine metabolites, plasma levels of KYN, 3-HK, AA, xanthurenic acid and quinolinic acid were still higher in uremic patients than those in healthy volunteers [55]. Uremic toxins from the kynurenine pathway like KYN and 3-HK are most frequent elevated in patients with CVD [56]. Moreover, tryptophan-derived uremic toxins from the indole pathway are also relevant to CVD in patients with CKD. IS was associated with the presence of CVD and cardiovascular mortality in uremic patients [57]. Moreover, bacterial tryptophan catabolites including IAA, IA, IAlD, ILA are AhR ligands [13]. Previous studies demonstrated that exogenous AhR ligand induces a high BP [58,59,60] and activation of AhR modulating T helper 17 (TH17) axis is involved in the development of hypertension [61]. Indoxyl sulfate (IS) is one of the most extensively studied uremic toxin. IS has been shown to induce inflammation and fibrosis in proximal tubule cells, impair the proliferation of endothelial cells, promote calcification of vascular smooth muscle cells, induce oxidative stress in proximal tubular and endothelial cells, and increase AhR-regulated gene expression in endothelial cells [57]. Together, these mechanisms are thought to cause CVD and CKD progression [57].

A growing body of evidence has demonstrated three tryptophan metabolic pathways connect hypertension and kidney disease (Figure 3), but relatively little is known about the role of maternal tryptophan metabolism in the development of hypertension and CKD in offspring. Whether targeting on tryptophan metabolic pathways can be applied in pregnancy to improve offspring renal outcomes remains to be addressed.

## 4. Tryptophan Metabolic Pathways: Programming versus Reprogramming Effects

### 4.1. Tryptophan-Related Metabolites-Induced Hypertension and CKD of Developmental Origin

A maternal low protein diet has been reported to program hypertension-related disorders in adult offspring in rodents, pigs, sheep, and cows [62,63,64]. Some studies demonstrated that maternal dietary tryptophan deficiency caused adverse effects on the development of the brain, liver, and skeletal muscle in rats [65,66,67]. However, there were no studies showing the programming effect of specific amino acid deficiency like tryptophan in pregnancy on offspring’s BP and renal outcome in humans and animals [28]. A plethora of tryptophan-derived metabolites have both detrimental and beneficial effects [11,12,13]. Therefore, excessive or deficit of a particular tryptophan-generating metabolite in pregnancy might be linked to hypertension and kidney disease in adult offspring. Maternal deficiency of melatonin, a tryptophan-derived metabolite produced in the serotonin pathway, has an increased risk for developing hypertension in adult offspring in a constant light exposure rat model [68]. Additionally, maternal CKD was reported to induce renal hypertrophy and hypertension in 12-week-old adult male rat offspring [69]. Since several uremic toxins from the kynurenine and indole pathways are endogenous ligands of AhR [58] and previous studies reporting maternal exposure to exogenous AhR ligand can induce hypertension and kidney disease in adult offspring [59,60], AhR activation might be an important target hub linking tryptophan metabolism and hypertension and kidney disease of developmental origin. Collectively, these observations suggest that dysregulated tryptophan metabolism in early-life is tightly linked to the risk for developing hypertension and kidney disease in adulthood.

### 4.2. Targeting on Tryptophan Metabolic Pathway as Reprogramming Strategies in Animal Models

Conversely, DOHaD theory offers a strategy to prevent the development of adult hypertension and kidney disease during early life, namely reprogramming [10]. Tryptophan supplementation has been used for the treatment of sleep disorders, pain, insomnia, depression, seasonal affective disorder, bulimia, attention deficit disorder, and chronic fatigue [11,70]. Nevertheless, less attention has been paid to study the potential beneficial effects of tryptophan supplementation during pregnancy and lactation on offspring health [11].

Since tryptophan-derived metabolites (e.g., serotonin and melatonin) and tryptophan-related signaling pathway (e.g., AhR) could be an alternative to obtaining the benefits provided by tryptophan, such tryptophan-related reprogramming interventions were recruited in the current review (Figure 3), with a focus on hypertension and kidney disease. We restricted this review to tryptophan-related interventions applied only during pregnancy or lactation periods, as there are critical periods for reprogramming strategies to prevent hypertension and kidney disease of developmental origin [71], which are listed in Table 1 [59,60,68,69,72,73,74,75,76,77,78,79,80,81,82].

Various adverse early-life environmental factors have been examined to induce hypertension and kidney disease in adult offspring, including a maternal CKD [69], SHR [72,80], a maternal caloric restriction [73], a maternal L-NAME exposure and/or postnatal high-fat diet [74,82], a maternal high-fructose diet [75], a maternal constant light exposure [68], a maternal high methyl-donor diet [76], a maternal high-fructose diet plus a post-weaning high-salt diet [77], a prenatal glucocorticoid (GC) exposure and/or post-weaning high-fat diet [78,79], a maternal plus post-weaning high-fructose diet [81], maternal 2,3,7,8-Tetrachlorodibenzo-p-dioxin (TCDD) and GC exposures [59], and a maternal bisphenol A (BPA) exposure and high-fat diet [60]. These diverse in utero insults cause adverse phenotypes in adult offspring including hypertension [68,69,72,73,74,75,76,77,78,79,80,81,82], altered transcriptome [76], and reduced nephron numbers [78]. All these adverse offspring outcomes can be prevented, or at least attenuated, by tryptophan-related interventions.

As shown in Table 1, rats are the most used subjects among animal models of developmental programming. One study showed tryptophan supplementation in pregnancy was reported to protect adult offspring against hypertension programmed by maternal CKD [69]. Although many researchers have studied tryptophan requirement in pigs [24], little is known whether tryptophan supplementation is beneficial in preventing kidney disease and hypertension in large animal models. Although a wide-range of metabolites come from tryptophan metabolism, only melatonin has been studied as a reprogramming intervention in pregnancy and lactation to protect adult offspring against hypertension and kidney disease of developmental origin [53]. Melatonin has pleiotropic biofunctions, such as antioxidant, anti-inflammation, regulation of circadian rhythm, and epigenetic regulation [53,83]; it also plays a vital role in pregnancy and fetal growth [84,85]. Perinatal melatonin therapy not only prevents hypertension programmed by diverse early-life insults [68,72,73,74,75,76,77,78,79] but also affects nephron number and renal transcriptome [76,78]. Reviews elsewhere have highlighted that low nephron number increases later life risk of hypertension and kidney disease [71,86]. In rats, prenatal glucocorticoid (GC) exposure causes a reduced nephron number and hypertension in adult offspring [78,87]. Melatonin therapy during pregnancy and lactation can prevent the reduction in nephron number and the rise of BP [78]. These findings indicate there is a renal reprogramming effect of melatonin protecting against maternal GC exposure-induced adverse offspring outcomes. Another study reported that a maternal methyl-donor diet results in alterations of renal transcriptome and programmed hypertension in adult rat offspring [76]. Conversely, maternal melatonin therapy altered 677 genes in renal transcriptome by which the elevation of BP in adult offspring can be attenuated [76]. Although oral tryptophan supplementation was reported to increase nocturnal circulating melatonin levels in Wistar rats [88], whether the above-mentioned beneficial effects of melatonin can be reached by maternal tryptophan supplementation remains to be elucidated. Like melatonin, serotonin is another important tryptophan-generating metabolite from the serotonin metabolic pathway. Being a neurotransmitter, the impact of serotonin has been extensively studied in developmental programming of neuropsychiatric disorders [34,89,90]. Nevertheless, its reprogramming effects on hypertension and kidney disease of developmental origin have not been reported yet.

On the other hand, several bacterial tryptophan catabolites from the indole and kynurenine pathways are AhR ligands [13]. Since maternal AhR activation is related to programmed hypertension and kidney disease in adult offspring [59,60], AhR antagonists might be a potential reprogramming strategy to reverse programming processes and prevent adverse outcomes. Resveratrol, a natural AhR antagonist [91], has been proposed to reprogram hypertension-related disorders [92]. This review presents that resveratrol supplementation in pregnancy and lactation can aid in preventing the development of hypertension in various developmental hypertension models, including SHRs [80], a maternal high-fructose diet [81], maternal TCDD and GC exposures [59], and a maternal BPA and high-fat diet [60]. So far, a specific AhR antagonist still remains inaccessible in clinical practice. Because resveratrol has multiple biological functions not just an AhR antagonist, whereas not all tryptophan-derived metabolites are AhR ligands, additional studies are required to elucidate which metabolite(s)-induced hypertension and kidney disease is AhR-dependent and develop a specific AhR-targeting approach as a reprogramming intervention in the future.

## 5. Common Mechanisms Link Tryptophan Metabolism to Developmental Programming of Hypertension and Kidney Disease

Although several organ systems are involved in the regulation of BP, renal programming is considered crucial in the development of hypertension and kidney disease [8,9,10]. It is clear from the preceding sections that diverse early-life environmental insults lead to same offspring phenotype (i.e., hypertension and kidney disease) indicating that there may be common mechanisms underlying renal programming. To date, animal models have provided insight on certain pathways underlying renal programming [8,9,10]. Notably, some of these mechanisms that have been previously connected to tryptophan metabolism include oxidative stress, gut microbiota, activation of the renin–angiotensin system (RAS), and immunity/inflammation. Each will be discussed in turn.

### 5.1. Oxidative Stress

Pregnancy is characterized by a state of high oxidative stress owing to increased basal oxygen consumption [93]. Oxidative stress is an imbalance between oxidants and antioxidants in favor of oxidants. Previous works that haven been published support that oxidative stress is important for the developmental programming of hypertension and kidney disease [71,94]. Various animal models demonstrate oxidative stress involved in renal programming and hypertension, including prenatal GC exposure [43], maternal caloric restriction [73], maternal high fructose diet [75], maternal high methyl-donor diet [76], maternal NO depletion [82], and maternal diabetes [95]. Several tryptophan-derived metabolites from the kynurenine pathway like kynurenine, 3-HK, 3-HAA, and quinolinic acid have shown pro-oxidant effects [96,97,98,99]. However, evidence regarding the antioxidant effects of metabolites generated from the kynurenine pathway have also been reported [100]. Additionally, it is acknowledged that uremic toxins from the indole metabolic pathway like IS and IDG can induce oxidative stress, which in turn contributes to the progression of CKD [23].

Conversely, antioxidant therapy in pregnancy has been shown to protect offspring against hypertension and kidney disease programmed by different in utero environmental insults [94,101]. Melatonin, a potent antioxidant from the serotonin pathway, has shown beneficial effects on hypertension and kidney disease of developmental origin [68,72,73,74,75,76,77,78,79]. Though there is evidence on the correlation between oxidative stress and tryptophan metabolism, a single unifying theory that can show the beneficial or detrimental effects of various metabolites generating from different pathways is lacking.

In a maternal CKD model, tryptophan supplementation during pregnancy and lactation protects offspring hypertension and is associated with restoration of nitric oxide (NO) [69]. NO is a vasodilator and free radical and plays a role in oxidative stress. NO deficiency and increased oxidative stress in the kidney contribute to the pathogenesis of hypertension [102]. Accordingly, targeting NO has been reported as a reprogramming strategy to prevent hypertension and kidney disease of developmental programming [102]. There is a close interlink between NO and tryptophan metabolism. NO can inhibit IDO activity [103], inactivate TPH [104], mediate melatonin production [105], and counteract the inhibitory effect of indole-derived uremic toxin IS [106]. The potential for use of tryptophan for its antioxidant properties in pregnancy must be investigated for its metabolism interplay with oxidative stress in determining its impact on hypertension and kidney disease of developmental origin.

### 5.2. Gut Microbiota

Dsybiotic gut microbiome in early life may have adverse effects resulting in adulthood diseases, including hypertension [107]. The role of gut microbiota in the pathogenesis of hypertension and CKD is suspected [108]. A growing body of evidence proposes several possible mechanisms to link gut dysbiosis and hypertension, including alterations of gut microbiota composition and their metabolites, increased sympathetic activity, activation of the renin–angiotensin system (RAS), and inhibition of NO [109]. Of note is that the composition of the microbiota determines several tryptophan metabolites as they are gut microbial catabolites. These tryptophan-derived microbial catabolites are crucial signaling molecules in host–microbial crosstalk contributing to systemic homeostasis [13]. Several bacterial species have been reported to produce tryptophan catabolites, such as *Clostridium, Bifidobacterium, Lactobacillus, Ruminococcus, Ruminiclostridium, Bacteroides*, and *Peptostretococcus* [13,110,111]. We recently reported that maternal tryptophan supplementation protects offspring against hypertension programmed by maternal CKD is associated with alterations to several tryptophan-metabolizing microbes, including *Lactobacillus*, *Ruminococcus*, and *Clostridium* [69]. The involvement of tryptophan-metabolizing microbes is obvious in terms of the ability to produce tryptophan catabolites but might also account for the pathogenesis of maternal CKD-induced hypertension.

In experimental and clinical CKD, microbiota-derived uremic toxins from indole and kynurenine pathways are increased and contribute to the progression of CKD and CVD [17,23,112]. Recent studies support the notion that microbiota-targeted therapies can be applied to a variety of diseases [113], including CKD [114,115]. Manipulating the gut microbiota with prebiotics or probiotics has been reported to reduce gut microbiota-derived uremic toxins in CKD [114,115]. In our hands, targeting gut microbiota by prebiotics (i.e., a special form of dietary fiber), probiotics (i.e., beneficial bacteria in the gut), or postbiotics (i.e., microbial metabolites) is able to prevent hypertension programmed by various early-life insults [116,117,118]. However, the identification of microbes involved in the modulation of tryptophan metabolite signaling and developing microbiota-targeted therapy for hypertension and kidney disease of developmental origin demand further investigation.

### 5.3. Renin–Angiotensin System

RAS is a coordinated hormonal cascade in the control of BP and renal physiology [119]. The classical RAS, defined as the angiotensin converting enzyme (ACE)–Ang II-angiotensin type 1 receptor (AT1R) axis, promotes sodium retention and elevation of BP. Conversely, the non-classical RAS composed of the ACE2–Ang-(1-7)-Mas receptor axis leads to vasodilatation [119]. Pharmacological blockade of the classical RAS is currently used to treat hypertension and kidney disease [120]. A growing body of evidence indicates that dysregulated RAS is a common mechanism underlying renal programming and programmed hypertension [6,8,9,10]. Early blockade of the classical RAS can reprogram inappropriate activation of the RAS and reverse the adverse programmed processes [121,122].

Several lines of observation show that the interplay between tryptophan metabolism and the RAS has an impact on renal programming and hypertension. First, several tryptophan-containing peptides have abilities to inhibit ACE activity and may serve as a potential anti-hypertensive therapy [123]. Second, activation of the kynurenine pathway is connected in parallel with the RAS in a renovascular hypertension model [45]. Third, there are studies showing that tryptophan-derived uremic toxin IS upregulate Ang II signaling and downregulate Mas, contributing to CVD and CKD [124,125]. Last, the preceding sections show that the maternal melatonin therapy which protects offspring against hypertension is, at least in part, attributed to the RAS in a maternal constant light exposure model [68], a maternal caloric restriction model [73], a maternal L-NAME exposure model [74], and a maternal high-fructose diet model [75].

In a maternal CKD model, adult male offspring-developed hypertension is related to decreased renal mRNA expression of ACE2, MAS, and AT2R, which belong to the non-classical RAS pathway [69]. Nevertheless, maternal tryptophan treatment prevented the elevation of BP but had neglectable effects on the RAS. Detailed mechanisms that underlie the interactions between tryptophan metabolic pathways and the RAS and their impact on the programming process toward hypertension, however, remain to be clarified.

### 5.4. Immunity and Inflammation

Pregnancy is characterized as a physiologic systemic inflammatory response; compromised pregnancies and related complications are associated with inflammation [126]. The interrelationship between inflammation and tryptophan metabolism has been reported in pigs, mice, and humans [127,128]. Hypertension and kidney disease are associated with the accumulation of T cells, monocyte/macrophages, and T cell–derived cytokines in the kidney [129]. An imbalance of T regulatory cells (Treg) and T helper 17 (TH17) cells has been associated with hypertension [130], which can be protected by restoration the balance of Treg/TH17 by postbiotic therapy [131]. In CKD, the interplay between Treg/TH17 balance and inflammation has also been associated with hypertension and the progression of CKD [132].

Given that both Treg and TH17 cells are regulated by AhR [133], and that several microbial tryptophan catabolites are AhR ligands, AhR can serve as a mediator in inflammation and CVD in patients with CKD [132]. AhR signaling can trigger inflammation via several mechanisms, including participating in T cell differentiation, increasing monocyte adhesion, up-regulating pro-inflammatory gene expression, inducing the expression of endothelial adhesion molecules, reducing NO bioavailability, and increasing endothelial cyclooxygenase-2 expression [23]. In a maternal CKD-induced hypertension model, the BP-lowering effect of tryptophan therapy is associated with mediation of the AhR signaling pathway [69]. Additionally, AhR antagonist resveratrol has been reported to protect offspring against hypertension in several developmental hypertension models [59,60,80,81]. However, more research is needed to gain comprehensive insight into the role of immunity and inflammation in the modulation of hypertension and kidney disease of developmental origin. Specifically, future studies should focus on an investigation of reprogramming intervention targeting of the mechanism of inflammation.

### 5.5. Others

There are other potential mechanisms which link tryptophan metabolism to the development programming of hypertension and kidney disease. First, epigenetic regulation such as DNA methylation, histone modification, and miRNAs altering the expression of genes has been considered as an important mechanism underlying renal programming [71]. Tryptophan metabolism has been identified as a significantly regulated Kyoto Encyclopedia of Genes and Genomes (KEGG) pathway in two-week-old (right after the completion of nephrogenesis) offspring kidneys in models of maternal caloric restriction and diabetes [134]. Thus, the genes involved in tryptophan metabolism are likely epigenetically regulated by early-life insults leading to programmed hypertension and kidney disease. Next, nutrient-sensing signals also play a role in renal programming [94]. NAD^+^ is a tryptophan metabolite generated from the kynurenine pathway. Increased NAD^+^/NADH ratio can activate silent information regulator transcript (SIRT) and cyclic adenosine monophosphate (AMP)-activated protein kinase (AMPK), consequently affecting PPARγ coactivator-1α (PGC-1α) activity to promote mitochondria biogenesis [135,136]. Since maternal resveratrol therapy protects hypertension programmed by maternal L-NAME plus postnatal high-fat exposure attributed to activation of the AMPK/PGC-1α pathway [82], whether tryptophan supplementation can increase NAD^+^ synthesis and activate nutrient-sensing signals deserves further evaluation.

## 6. Conclusions

Although substantial progress has been made in understanding the role of tryptophan metabolism in pregnancy and offspring outcomes, there is always more to learn. Given tryptophan produces a plethora of biologically active metabolites, deciphering the complexity of different tryptophan metabolic pathways will aid in developing ideal reprogramming strategies targeting different tryptophan-related elements to open therapeutic opportunities for clinical translation. This review has provided an overview on reprogramming strategies against hypertension and kidney disease excepting tryptophan, which are related to the tryptophan metabolism, including melatonin and the AhR antagonist. Further research is needed to gain a clear understanding of the type of tryptophan-related molecules, the therapeutic dose and duration in pregnancy, and the microbial groups to metabolize tryptophan before the mother and child can benefit from reprogramming strategies targeting the tryptophan metabolism.

## Figures and Tables

**Table 1:** Reprogramming effects protect adult offspring against hypertension and kidney disease by tryptophan-related interventions.

Interventions	Animal Models	Species/Gender	Age at Measure	Reprogramming Effects
Tryptophan				
Tryptophan 200 mg/kg BW/day via oral gavage during pregnancy	Maternal adenosine-induced CKD	SD rat/M	12 weeks	Prevented hypertension [69]
Melatonin				
10 mg/kg BW/day melatonin in drinking water during pregnancy	Genetic hypertension model	SHR/M	16 weeks	Prevented hypertension [72]
0.01% melatonin in drinking water during pregnancy and lactation	Maternal caloric restriction	SD rat/M	12 weeks	Prevented hypertension [73]
0.01% melatonin in drinking water during pregnancy and lactation	Maternal L-NAME exposure	SD rat/M	12 weeks	Prevented hypertension [74]
0.01% melatonin in drinking water during pregnancy and lactation	Maternal high-fructose diet	SD rat/M	12 weeks	Prevented hypertension [75]
0.01% melatonin in drinking water during pregnancy and lactation	Maternal constant light exposure	SD rat/M	12 weeks	Prevented hypertension [68]
0.01% melatonin in drinking water during pregnancy and lactation	Maternal methyl-donor diet	SD rat/M	12 weeks	Attenuated hypertension and altered renal transcriptome [76]
0.01% melatonin in drinking water during pregnancy and lactation	Maternal high-fructose diet plus post-weaning high-salt diet	SD rat/M	12 weeks	Attenuated hypertension [77]
0.01% melatonin in drinking water during pregnancy and lactation	Prenatal GC exposure	SD rat/M	16 weeks	Prevented hypertension and increased nephron number [78]
0.01% melatonin in drinking water during pregnancy and lactation	Prenatal GC exposure plus post-weaning high-fat diet	SD rat/M	16 weeks	Prevented hypertension [79]
AhR antagonist				
4 g/kg diet resveratrol during pregnancy and lactation	Genetic hypertension model	SHR/M and F	20 weeks	Prevented hypertension [80]
50 mg/L resveratrol in drinking water during pregnancy and lactation	Maternal plus post-weaning high-fructose diet	SD rat/M	12 weeks	Prevented hypertension [81]
0.05% resveratrol in drinking water during pregnancy and lactation	Maternal TCDD and GC exposures	SD rat/M	16 weeks	Prevented hypertension [59]
50 mg/L resveratrol in drinking water during pregnancy and lactation	Maternal bisphenol A exposure and high-fat diet	SD rat/M	16 weeks	Prevented hypertension [60]
50 mg/L resveratrol in drinking water during pregnancy and lactation	Maternal L-NAME plus postnatal high-fat diet	SD rat/M	16 weeks	Prevented hypertension [82]

CKD = chronic kidney disease; SD = Sprague–Dawley; M = male; F = female; SHR = spontaneously hypertensive rat; L-NAM E = N^G^-nitro-L-arginine methyl ester. GC = glucocorticoid; AhR = aryl hydrocarbon receptor; TCDD = 2,3,7,8-Tetrachlorodibenzo-p-dioxin.

## Abbreviations

AANAT	Arylalkylamine N-acetyltransferase
ACE	Angiotensin converting enzyme
AhR	Aryl hydrocarbon receptor
AMPK	Adenosine monophosphate activated protein kinase
ArAT	Acromatic amino acid aminotransferase
ASMT	N-acetylserotonin O-methyltransferase
AT1R	Angiotensin type 1 receptor
AT2R	Angiotensin type 2 receptor
CKD	Chronic kidney disease
CVD	Cardiovascular disease
NAD^+^	Nicotinamide adenine dinucleotide
DOHaD	Developmental origins of health and disease
GC	Glucocorticoid
IAA	Indoleacetic acid
IAM	Indole-3-acetamide
IAlD	Indole-3-aldehyde
IDG	Indoxyl-β-D glucuronide
IDO	Indoleamine 2,3-dioxygenase
ILA	Indolelactic acid
IPA	Indole-3-propionic acid
KAT	Kynurenine aminotransferase
KMO	Kynurenine-3-monooxygenase
KYNU	Kynureninase
L-NAME	N^G^-nitro-l-arginine-methyester
MAO	monoamine oxidase
Mas	Angiotensin-(1-7) receptor
NO	Nitric oxide
PGC-1α	PPARγ coactivator-1α
RAS	Renin-angiotensin system
ROS	Reactive oxygen species
SHR	Spontaneously hypertensive rat
SIRT	Silent information regulator transcript
SSRI	Selective serotonin reuptake inhibitor
TCDD	2,3,7,8-Tetrachlorodibenzo-p-dioxin
TDO	Tryptophan 2,3-dioxygenase
TPH	Tryptophan hydroxylase
3-HK	3-hydroxykynurenine
5-HIAA	5-hydroxyindoleacetic acid
5-HTP	5-hydroxytryptophan
